# Assessing the Influence of Low Doses of Sucrose on Memory Deficits in Fish Exposed to Common Insecticide Based on Fipronil and Pyriproxyfen

**DOI:** 10.3390/cimb46120848

**Published:** 2024-12-15

**Authors:** Viorica Rarinca, Luminita Diana Hritcu, Marian Burducea, Gabriel Plavan, Radu Lefter, Vasile Burlui, Laura Romila, Alin Ciobică, Elena Todirascu-Ciornea, Cristian-Alin Barbacariu

**Affiliations:** 1 Doctoral School of Geosciences, Faculty of Geography and Geology, “Alexandru Ioan Cuza” University of Iasi, No 20A, Carol I Avenue, 700505 Iasi, Romania; rarinca_viorica@yahoo.com; 2 Doctoral School of Biology, Faculty of Biology, “Alexandru Ioan Cuza” University of Iași, Carol I Avenue, 20A, 700505 Iasi, Romania; 3 Preclinical Department, Apollonia University, Pacurari Street 11, 700511 Iasi, Romania; vburlui@gmail.com; 4 Department of Public Health, Faculty of Veterinary Medicine, Iasi University of Life Sciences, Mihail Sadoveanu Street, No. 3, 700490 Iasi, Romania; lumidih@yahoo.com; 5 Research and Development Station for Aquaculture and Aquatic Ecology, “Alexandru Ioan Cuza” University, Carol I, 20A, 700505 Iasi, Romania; marian.burducea@uaic.ro (M.B.); alin.barbacariu@uaic.ro (C.-A.B.); 6 Department of Biology, Faculty of Biology, Alexandru Ioan Cuza University of Iasi, No 20A, Carol I Avenue, 700505 Iasi, Romania; gabriel.plavan@uaic.ro (G.P.); ciornea@uaic.ro (E.T.-C.); 7 Center of Biomedical Research, Romanian Academy, No. 8, Carol I Avenue, 700506 Iasi, Romania; radu_lefter@yahoo.com; 8 Academy of Romanian Scientists, No. 54, Independence Street, Sector 5, 050094 Bucharest, Romania

**Keywords:** sucrose, common insecticide, fipronil, pyriproxyfen, memory deficits, Silver crucian carp

## Abstract

Although pesticides have been a constant concern for decades, in the last ten years, public discussions and scientific research have emphasized their impact on human health and the environment, drawing increased attention to the problems associated with their use. The association of environmental stressors such as pesticides with a sugar-rich diet can contribute to the growing global metabolic disease epidemic through overlapping mechanisms of insulin resistance, inflammation, and metabolic dysregulation. The main aim of this study was to evaluate the behavioral effects of the exposure of Silver crucian carp (*Carassius auratus gibelio*) to a commercial insecticide formulation containing fipronil, pyriproxyfen, and other additives, as well as sucrose and their mixtures. The behavioral responses in the T-test showed significant abnormalities in the exploratory activity evocative of memory deficits and an increased degree of anxiety in the groups of fish treated with the insecticide formulation and the mixture of the insecticide with sucrose. Aggression, quantified in the mirror-biting test, as biting and the frequency of approaches to the mirror contact zone, was significantly decreased only in the insecticide and sucrose group. All three groups showed behavioral changes reflective of toxicity, but only the combination of the two stress factors, environmental (insecticide) and metabolic (sucrose intake), resulted in pronounced memory alterations.

## 1. Introduction

Insecticides are chemical agents used to kill or control insects, playing a crucial role in agriculture and pest management. One notable insecticide is fipronil (5-amino-1-(2,6 dichloro-α,α,α-trifluoro-p-tolyl)-4-trifluoromethyl sulfinyl pyrazole-3-carbonitrile), which is a widely used broad-spectrum phenylpyrazole insecticide and an active ingredient of numerous registered products designed for agricultural or veterinary pests [1]. Its efficacy in combating certain pests, such as lepidopterans, orthopterans, and others, which can develop resistance to other commonly used insecticides, is due to its selective mechanisms of action [2,3,4]. The insecticide causes toxicity by disrupting the opening of γ-aminobutyric acid (GABA)-mediated chloride channels, which leads to nervous hyper-excitation, paralysis, and death in the context of the unchecked excitatory effects of sodium ions [4,5]. Exposure to fipronil is reported to alter metabolic enzyme systems containing sulfhydryl groups, as well as mitochondrial oxidative phosphorylation [6]. Decreased antioxidant capacity in all organs [7] and subsequent liver and kidney histopathological alterations, pulmonary toxicity [8,9], and apoptosis in neuronal cell lines [6] have been reported. The changes in the levels of oxidative stress indicators, such as superoxide dismutase (SOD), glutathione peroxidase (GPx), and malondialdehyde (MDA), reflecting the accumulation of ROS, coupled with unusual increases in liver enzymes and hepatocyte hypertrophy [10], could provide a relevant test for assessing the impact of fipronil exposure in various species [7].

Very few studies have explored the effects of fipronil exposure on memory, given the relevance of GABA neurotransmission in memory processes, specifically spatial working memory [11] and fear memory [12]. A pilot experiment assessing the central GABAergic system’s role in cognition reported that short-term exposure to fipronil significantly interferes with performance in cognitive and spatial memory tasks and that the memory alterations are further enhanced by co-exposure to the GABA antagonist picrotoxin [13]. Similarly, impaired olfactory learning and memory following exposure to very low doses of fipronil were described in the honeybee as linked to the high sensitivity of ionotropic GABA receptors to the insecticide [14]. Other behavioral perturbations such as hyperlocomotion or changes in motor activity and coordination were noted when fipronil was either directly injected into the substantia nigra of male rats [15] or under sustained long-term exposure in female mice [16].

Sucrose (C_12_H_22_O_11_), also known as food sugar, is a disaccharide resulting from the binding of two monosaccharides, glucose and fructose, using a glycosidic bond [17]. Sucrose is a rapidly assimilated macronutrient that provides quick energy [18], but, in excess, is associated with metabolic disorder such as cardiovascular disease, type 2 diabetes, and obesity [19,20]. Studies in rodent and zebrafish models reveal links between high-sucrose diets, oxidative stress, and metabolic disturbances, offering insights into similar impacts on aquatic organisms [21,22,23,24,25,26]. In a recent review, the hyperglycemiant regime was linked to the installment of neurological deficits, brain elevated levels of pro-inflammatory molecules, and disturbances along the HPA axis co-occurring with behavioral alterations evoking anxiety-like behaviors, cognitive deficits, and social stress responses [27]. Cognitive alterations and the onset of Alzheimer’s disease (AD) are linked to one another in many epidemiological studies [23,28,29], as well as to pathological cerebral glucose metabolic alterations including glucose metabolism dysregulation, glycolysis dysfunction, and pentose phosphate pathway impairment, which are also oxidative stress pathways [30]. A large epidemiological study with over 18 years of data reported that excessive total sugar intake is significantly associated with AD risk in women [31]. Additionally, the theory of Alzheimer’s as type 3 diabetes, characterized by pronounced insulin resistance within the brain, suggests a potential cause for compromised amyloid-β processing and clearance [32].

Pesticide exposure, such as organophosphate (OP), pyrethroid (PYR), and organochlorine (OC), and high-sucrose diets are linked to increased risks of developing metabolic disorders such as insulin resistance (IR), diabetes, and obesity [33,34,35,36]. Studies have found associations between pesticide exposure and higher rates of diabetes, obesity, hypertension, chronic kidney disease, and metabolic syndrome [33]. Furthermore, understanding how these factors affect aquatic organisms provide insights into broader ecological impacts. The combination of pesticide exposure and a high-sucrose diet appears to have a synergistic effect, increasing the risk of developing metabolic disorders [34]. Pesticides can disrupt normal metabolic processes, while a high-sugar diet provides the substrate to exacerbate these disruptions, ultimately driving the development of insulin resistance, obesity, and related conditions [33]. Both stressors contribute to the global epidemic of metabolic diseases through overlapping mechanisms of insulin resistance, inflammation, and metabolic dysregulation [33,34,35,36].

More researchers are recognizing that there is a significant connection between AD and metabolic syndrome, as a strong association between the two conditions has been observed. Studies have shown that people with metabolic syndrome have an increased risk of developing AD [37,38,39]. Specifically, metabolic syndrome, characterized by components such as abdominal obesity, hypertension, hyperglycemia, and dyslipidemia, has been associated with a higher likelihood of AD [40]. This association is supported by findings showing that metabolic syndrome contributes to cognitive impairment in the elderly and correlates with an increased risk of type 2 diabetes, a condition which has links to AD [38,41]. In addition, factors such as IR, high plasma triglycerides, and low plasma HDL cholesterol, which are common in metabolic syndrome, have been implicated in the development and progression of Alzheimer’s disease [41]. Overall, the relationship between AD and metabolic syndrome underscores the importance of understanding how metabolic factors may affect cognitive health and increase risks for neurodegenerative disorders. Alzheimer’s being termed “type 3 diabetes” suggests a link between insulin resistance and cognitive decline [42]. This concept underscores the intricate connection between metabolic health and brain function [43]. Exploring these metabolic factors could lead to novel treatment approaches targeting both diabetes and AD, offering new hope for combating these interconnected conditions.

Carp are widely recognized in aquatic toxicology and neuroscience research due to their well-characterized physiology and behavior and their relevance for studying environmental impacts on aquatic ecosystems. This makes them suitable for studying neurotoxic effects and cognitive functions [44]. In addition, it represents a relevant model for assessing the impact of environmental contaminants on aquatic organisms. While traditional mammalian models are more prevalent, there is a growing body of research exploring neurodegenerative processes in fish, including carp [44,45]. Recent studies have indicated that certain behavioral and cognitive disturbances observed in fish may parallel aspects of neurodegenerative diseases such as Alzheimer’s, particularly when exposed to neurotoxicants [44].

In the present study, we evaluated the impact of acute exposure on memory and behavioral parameters in Silver crucian carp. We also investigated whether this impact could be enhanced by concurrent acute exposure to a medium/high concentration of sucrose. By exploring the combined effects of insecticide and sucrose, we aimed to understand the interaction between environmental contaminants and dietary factors on cognitive and behavioral functions, with implications for human health. The novelty of this study lies in examining the combined impact of insecticide exposure and sucrose intake on cognitive and behavioral functions, providing insights into how these environmental and dietary factors may interact to affect neurobehavioral outcomes.

## 2. Materials and Methods

### 2.1. Animals

For this experiment, *C. auratus gibelio* seedlings were acquired from the Aquaculture and Aquatic Ecology Research—Development Station (Ezăreni, Iași), with an average weight of 5 ± 1 gr and an average length of 4 ± 0.5 cm. The fish were housed in 10 L glass aquaria with adequate aeration and filtration for one week from their arrival. The quality parameters of the water were checked from the first day housing the fish, obtaining the following values: temperature 23 °C, pH 7.07, and water strength of 314 mg/L CaCO_3_. The fish were maintained under artificial lighting conditions of 12 h light with 12 h dark and fed special fish food (flake food) twice per day.

Throughout the duration of the acclimation period and subsequent treatments, the fish were monitored for any clinical signs of toxicity. Observations confirmed that all fish appeared healthy, with no overt signs of distress or illness noted. Additionally, the fish were observed to be feeding normally during the study period.

The animals were treated in accordance with the animal bioethics regulations of the Animal Experimentation Law and the Romanian Animal Health and Welfare Law, and all procedures complied with Directive 2010/63/EU of the European Parliament and the Council of 22 September 2010 on the protection of animals used for scientific purposes. Every effort was made to reduce the suffering and number of animals in the experiments. The experiment was approved by the Ethical Commission of the Faculty of Veterinary Medicine, University of Life Sciences Iasi, with registration number 748/04.07.2019.

### 2.2. Experimental Design

In the current study, we used 4 groups of fish, *n* = 14 individuals/group, as follows: Group 1 was the control group, which remained untreated during the three days of treatment; Group 2 received 28.44 g/mL sucrose; Group 3 was exposed to 100 μg/L insecticide; and Group 4 co-treated with insecticide and sucrose.

The sucrose exposure treatment was administered over a 3-day period using a sucrose concentration of 28.44 g/mL, adapted from Ranjan and Sharma (2020) [46]. Exposure took place in an aquarium, using commercial food sugar diluted according to the concentration mentioned above, in a sufficient volume of water taken from the fish aquarium. The obtained solution was returned to the fish aquarium so that the water volume remained the original 10 L. The treatment was renewed every 24 h over the three days. The insecticide treatment was administered for three days and consisted in the administration of insecticide (purchased from the Faculty of Veterinary Medicine, University of Life Sciences of Iasi, Romania) directly in aquarium water at a concentration of 100 μg/L (adapted from [47,48,49]). The concentration used was relatively not very high compared to the probable values that can appear in the environment and was located in the lower half of the lethality scale, reported at 246 μg/L [50], which would favor, theoretically, the induction of toxic effects over a short period, without inducing lethality. The dose calculated to ensure the mentioned concentration within the volume of 10 L of the aquarium was renewed every 24 h. For Group 4, treatment was administered with insecticide and sucrose, using the same administration methods: the diluted sugar solution and insecticide were administered at the same time intervals; the treatment was also renewed every 24 h. Behavioral tests were performed over 4 days following the end of the exposure period, between 10:00 AM and 4:00 PM. At the end of this stage, 6 individuals from each group were sacrificed by immersion in ice water and frozen for subsequent oxidative stress analyses.

### 2.3. Behavioral Testing

The spontaneous exploration test was performed in a T-shaped device made of semi-transparent plexiglass (20 × 50 × 50 cm/height × length × width) and filled ¾ with water. The method used was adapted from Grossman et al. (2010), Zizza et al. (2017), Cleal et al. (2021) [51,52,53]. The animals were individually introduced at the end of the long arm and allowed to freely explore the apparatus for 6 min without any prior habituation. The paradigm was based on the animal’s exploratory tendency and spatial short-term memory, similar to the Y-test used in rodents but with some fundamental changes. Thus, we conducted this experiment as described by the group of Parker from University of Portsmouth, UK, in several papers published starting in 2019 [54]. The main behavioral indicator we referred to for the short-term working memory was the motor strategy adopted by the fish, visible in the sequences of turns to the left or the right in the decisional point of the maze, not in the arm entry successions, as used in rodents. These sequences of left and right turns were apportioned in sixteen possible tetragrams (combinations of four turns), out of which only the right–left–right–left (RLRL) and left–right–left–right (LRLR) tetragrams or the alternation tetragrams—differentiated from the other “repetition” tetragrams—represent the exploration pattern relevant to the memory-based search strategy in fish [53]. The behavioral parameters were manually assessed during the test and included the following: latency time required to enter one of the side arms; the number of turns and the percentage of the two specific alternation tetragrams (RLRL and LRLR) and the time spent in each of the arms; and the number of freezing episodes (state of total immobility) and their duration.

The light/dark preference test for anxiety assessment was adapted from [51,52,55]. A rectangular glass apparatus (30 × 20 × 20 cm/length × width × height) filled with water up to ¾ of its capacity and divided into two equal compartments, demarcated by dark and light illumination, but without a physical separation, was used. Dark illumination was provided by covering the side walls and the top of the aquarium with opaque cardboard, and the lit side was left exposed above and on the sides, except for the bottom and the wall corresponding to the width, which were covered with a white sheet to cancel the light reflection and tendency of fish to interact with their reflected image. Lighting was constant and provided artificially from above with a dim neon light. Each fish was individually introduced into the dark half of the aquarium and allowed to freely explore the apparatus for 6 min, during which it was videotaped. The behavioral indicators analyzed included the following: latency of entering the illuminated compartment; the time spent in the illuminated compartment; the number of passages between the two compartments; and the risk-assessment behavior, represented by the number of burstings (fast <1 s entries in the white compartment followed by re-entry into the dark compartment) and partial entries into the light compartment with full return angles.

The mirror-biting test for evaluating the manifestations of aggressiveness in a fish towards its reflection in a mirror was conducted in a standard rectangular glass apparatus (30 × 10 × 20 cm/length × width × height). A 4.5 × 3.8 cm mirror was placed on one of the side edges of the aquarium (left) at an angle of 22.5° to the back wall. The front face of the apparatus was delimited into 4 equal quadrants, the mirror being positioned in the lower left quadrant, so that it was possible to count the entries in each sector. Each fish was introduced into the aquarium and allowed to freely explore the apparatus for 6 min. Entries in the lower left sector were representative of a preference for proximity/confrontation with a rival, while entries in the other segments reflected an avoidance attitude. Two virtual demarcations were considered for further behavioral analysis: the direct contact zone, up to 0.5 cm from the mirror, and the proximity zone, up to 2.5 cm from the first line. The behavioral parameters video-recorded were the following: the mirror-biting frequency and duration; the mirror approach frequency (without contact with the mirror); contact latency; and other behavioral parameters reflecting the appearance of the state of anxiety, such as the duration of freezing.

### 2.4. Oxidative Stress Analysis

For the biochemistry analyses, fish brain tissues (from 5 specimens per experimental group) were collected, well homogenized in 10 volumes of ice-cold saline (0.90% NaCl), and centrifuged at 5500 rpm for 10 min at 4 °C according to a previous protocol [47]. The supernatant of each sample was transferred to a clean PE tube and divided into aliquots for total protein analysis and SOD, GPx, catalase, carbonylated protein, acetylcholinesterase (ACHE), and MDA measurements. The SOD, GPx, catalase, carbonylated protein, acetylcholinesterase ACHE, and MDA levels, as well as the protein concentrations of the tissue suspensions, were determined using the Merck assay kits and protocols. A spectrophotometer Specord 210 Plus producer Analytik Jen (Jena, Germany) was used to determine total protein, SOD, GPx, catalase, carbonylated proteins, ACHE, and MDA. Data were expressed as the average ± SD.

### 2.5. Chemicals

Physiological saline (0.90% NaCl) was purchased from Hemofarm (Timisoara, Romania). We used a common insecticide purchased from the Faculty of Veterinary Medicine, University of Life Sciences, with a quality certificate and soluble in water. Every 1.5 mL of this insecticide had the following composition: 67.5 mg of fipronil, 67.5 mg of pyriproxyfen, 0.3 mg of butylated hydroxyanisole, 0.15 mg of butylhydroxytoluene, and 60 mg of benzyl alcohol. For the OS, the following related kits were used: Superoxide Dismutase Determination Kit (SOD, 19160-1KIT-F), Lipid Peroxidation (MDA) Test Kit (MAK085-1KIT), Cell Activity Test Kit for Glutathione Peroxidase (GPx, CGP1-1KIT), Protein Carbonyl Content Assay Kit (MAK094-1KIT), and Catalase (CAT, 219265-1KIT) were purchased from Merck, Darmstadt, Germany. The SOD Assay Kit quantified the activity of superoxide dismutase, an enzyme which protects cells from damage by converting superoxide radicals into less-harmful molecules. This assay involved mixing samples with a substrate and measuring changes in absorbance to determine enzymatic activity. Next, we used the MDA Assay Kit to measure malondialdehyde, a byproduct of lipid peroxidation which indicates cell membrane damage. In this test, samples were mixed with reagents and heated to form a colored complex, which was then quantified using absorbance measurements. We also used the GPx Cellular Activity Assay Kit to assess the activity of glutathione peroxidase, another important antioxidant enzyme. This test involved incubating samples with specific substrates and measuring the resulting reaction spectrophotometrically. In addition, we used the Protein Carbonyl Content Assay Kit to assess protein carbonyls, which are markers of oxidative stress and protein damage. This kit required treating samples with reagents that reacted to form a colored product, allowing quantification. Finally, the CAT Kit was used to measure the activity of catalase, which breaks down hydrogen peroxide into water and oxygen. The protocol consisted of adding samples to a reaction mixture and monitoring the decrease in hydrogen peroxide concentration over time.

### 2.6. Statistical Analysis

The numerical data obtained via the behavioral tests were statistically analyzed by using a one-way analysis of variance (ANOVA) and Tukey’s multiple comparisons of means (Minitab 19 software (Minitab Inc., 2019). All results were expressed as the mean ± SEM. Post hoc analyses were performed using Tukey’s honestly significant difference test in order to compare groups. F values for which *p* was < 0.05 were regarded as statistically significant.

## 3. Results

### 3.1. T-Maze Test—Evaluation of Spontaneous Exploratory Behavior and Short-Term Memory

**Figure 1:**
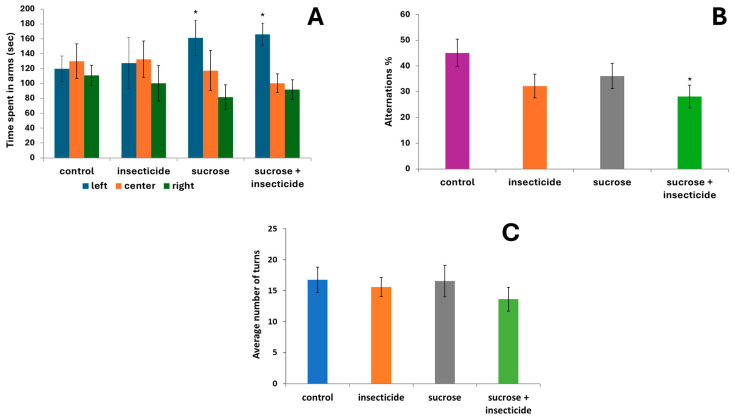
Exploratory behavior and short-term memory assessment in the T-maze task after acute exposure to sucrose, insecticide, and sucrose + insecticide: (**A**) time spent (s) in each of the three arms of the maze; (**B**) total number of entries in the arms of the maze; and (**C**) the effects on short-term memory assessed by the succession of arm entries after acute exposure to sucrose, insecticide, and sucrose + insecticide. Data are expressed as the average ± SD (*n* = 14, * *p* < 0.05 vs. control).

Three important parameters were evaluated in the T-maze test: time spent (s) and number of turns during the task, reflecting exploratory and motor activity, and the alternation tetragrams based on the turning sequences, relevant for short-term spatial memory. For the time spent in the three arms, as shown in Figure 1A, we observed a relatively uniform exploration for the control group and the sucrose group, and a significant preference to explore only one of the arms (left arm) over the other two in the insecticide group vs. control (161.07 ± 23.68 vs. 119.53 ± 17.33, *p* < 0.05) and in the combined sucrose + insecticide group (166 ± 14.78 vs. 119.53 ± 17.33, *p* < 0.05 vs. control).

Exploratory and locomotory activity expressed as the total number of entries in the arms (Figure 1B) did not significantly differ between the groups (*p* = 0.6). The tetragram analysis revealed a significant decrease in the percentage of alternating tetragram sequences (LRLR, RLRL), in the sucrose + insecticide group vs. control (28.12 ± 4.37 vs. 45.2 ± 5.31, *p* < 0.02). The analysis of the percentage of alternating tetragrams also showed a decrease in the insecticide group vs. the control group, but this was not statistically significant (32.24 ± 4.65 vs. 45.2 ± 5.31, *p* = 0.081). These results are suggestive of the appearance of short-term memory disturbances in the sucrose + insecticide group (Figure 1C).

### 3.2. Light/Dark Preference Test—Anxiety Assessment

**Figure 2:**
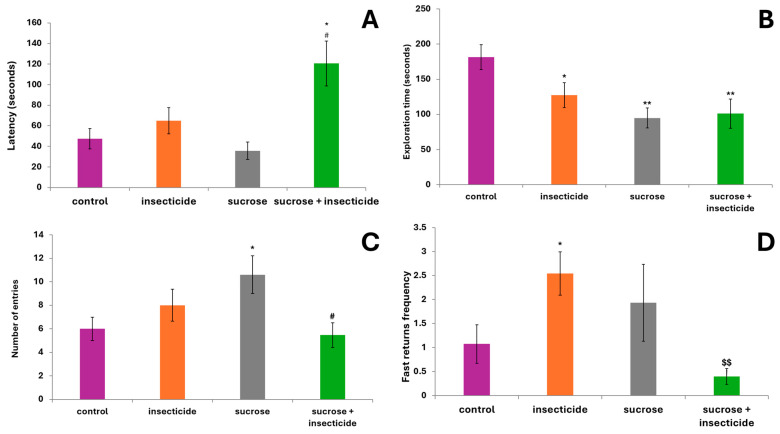
Behavioral assessment in the light/dark preference test after acute exposure to sucrose, insecticide, and sucrose + insecticide: (**A**) latency time (s) until the first entry in the lit compartment; (**B**) time spent (s) in the lit compartment of the apparatus; (**C**) number of entries into the lit compartment; and (**D**) frequency of risk-appraisal behaviors, represented by the number of fast returns into the dark compartment and partial entries into the lit compartment. Data are expressed as the average± SD (*n* = 14, * *p* < 0.05 vs. control, ** *p* < 0.01 vs. control, # *p* < 0.05 vs. sucrose, and $$ *p* < 0.01 vs. insecticide).

The latency to enter the lit compartment, which was considered a more exposed area compared to the dark half of the compartment, was significantly increased only in the sucrose + insecticide group, which started exploring the apparatus after twice the time compared to the control (47.3 ± 9.80 vs. 120.6 ± 21.8, *p* < 0.05) or the sucrose group (35.6 ± 8.4 vs. 120.6 ± 21.8, *p* < 0.01) (Figure 2A).

Regarding the time spent (s) in the lit compartment of the device, the main parameter for assessing the anxiety states, all three treated groups explored this area significantly less compared to the control group. The insecticide group spent, on average, a third less time in the exposed area than the control group (127.5 ± 17.6 vs. 181.5 ± 17.7, *p* < 0.05 vs. control). The sucrose and sucrose + insecticide groups explored the lit compartment half as long as the control: 94.9 ± 14.1 vs. 181.5 ± 17.7, *p* < 0.01 vs. control, and 10 ± 20.59 vs. 181.5 ± 17.7, *p* < 0.01 vs. control, respectively (Figure 2B).

Regarding the number of entries into the illuminated compartment of the apparatus, while the insecticide group and sucrose + insecticide group did not show significant differences compared to the control, the sucrose group had a significantly higher number of entries compared to the control (10.6 ± 1.61 vs. 6 ± 0.99, *p* < 0.05). This result was more relevant to a state of hyperactivity after exposure to sucrose than to an anxiolytic state, as it was not correlated with the reduced duration of stay in the lit compartment. Exposure to the combination of sucrose and insecticide significantly reduced the number of entries compared to the sucrose group (5.46 ± 1.045 vs. 10.6 ± 1.61, *p* < 0.05 vs. sucrose group) (Figure 2C).

The risk-assessment behavior consisting in rapid withdrawals into the dark compartment and only partial entries into the lit compartment varied between the groups (Figure 2D). The insecticide group displayed consistently extremely high values of burstings and fast returns compared to the control group (2.54 ± 0.45 vs. 1.07 ± 0.4, *p* < 0.05), suggestive of increased anxiety states. The risk-assessment manifestations in the sucrose group were also increased compared to the control, but they were under the significance threshold. Interestingly, the number of risk-appraisal behaviors recorded in the group of fish treated with sucrose combined with insecticide was, on average, lower than all other groups and significantly reduced compared to the insecticide group (0.4 ± 0.16 vs. 2.54 ± 0.45 *p* < 0.01). Considering the exceptionally low number of entries into the lit area recorded among fish from the group treated with sucrose and insecticide, the low frequency of risk-assessment behaviors could have been caused by a lack of exposure to the unknown environment.

### 3.3. Mirror-Biting Test

**Figure 3:**
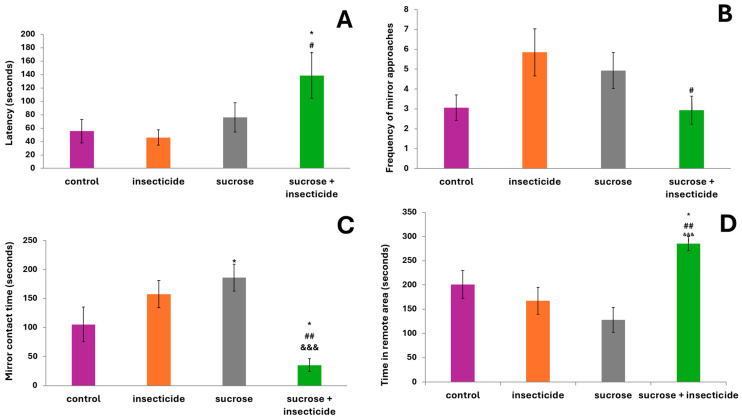
Assessment of aggressiveness level in the mirror-biting test after acute exposure to sucrose, insecticide, and sucrose + insecticide: (**A**) latency time (s); (**B**) number of entries into the mirror contact zone; (**C**) mirror contact duration (s); and (**D**) frequency of risk-appraisal behaviors, represented by the number of fast returns to the dark compartment and partial entries into the lit compartment. Data were expressed as the average± SD (*n* = 14, * *p* < 0.05 vs. control, # *p* < 0.05 vs. insecticide, ## *p* < 0.01 vs. insecticide, and &&& *p* < 0.001 vs. sucrose).

For this test, the latency to enter in the mirror contact zone, an indicator of anxiety, showed a similar pattern to the results obtained in the light/dark test, with only the sucrose + insecticide group showing a significantly increased delay in entering the target area compared to the control (138.4 ± 34.12 vs. 55.42 ± 17.3, *p* < 0.05) and the insecticide group (138.4 ± 34.12 vs. 46.05 ± 11.35, *p* < 0.05) (Figure 3A).

Regarding the frequency of entries into the mirror contact zone, where the test individual faces its own reflection in the mirror, interacting with it as if it were a conspecific rival, no significant differences appeared between the groups. However, the number of entries in the contact zone (the area next to the mirror) for the insecticide group was, on average, higher than the control (5.8 ± 1.1 vs. 3.06 ± 0.64, *p* = 0.06 vs. control). A marked reduction in the frequency of entries in the contact zone was observed for the group treated with sucrose and insecticide, significantly lower than the insecticide group (2.93 ± 0.7 vs. 5.8 ± 1.1, *p* < 0.05). This decreased tendency toward aggressiveness in the group treated with sucrose and insecticide correlated inversely with the high values of anxiety in the previous test (Figure 3B).

One of the most important parameters measured in the aggressiveness test is the length of time spent in the area of contact with the mirror, during which the individual exhibits various dominant and aggressive behaviors, such as attacking the mirror, biting the reflection, or showing off on the side. Increased aggressiveness was observed in the sucrose group, which spent significantly more time exhibiting aggressive behavior in front of the mirror (186.13 ± 23.3 vs. 105.2 ± 29.8, *p* < 0.05 vs. control). The insecticide group spent, on average, a longer time in the mirror contact area, but this increase was not statistically significant relative to the control. Interestingly, the sucrose + insecticide group spent significantly less time in the contact zone compared to all other groups: 35.2 ± 10.8 vs. 105.2 ± 29.8, *p* < 0.05 vs. control; 35.2 ± 10.8 vs. 157.5 ± 23.4, *p* < 0.01 vs. insecticide; and 35.2 ± 10.8 vs. 186.13 ± 23.3, *p* <0.001 vs. sucrose. Corroborated with the other data, these data could suggest the prevalence of aggressive states and anxiety in the case of the sucrose group and sucrose + insecticide group, respectively (Figure 3C).

We also observed the instillation of significant freezing durations in the insecticide group (*p* = 0.033 vs. control), in which approximately 1/3 of the total number of tested individuals exhibited prolonged immobility during the six-minute test. Given that no individual in the sucrose + insecticide group entered states of total immobility, a question arose as to whether sucrose in low concentrations had a beneficial effect in counteracting insecticide-induced anxiety.

The analysis of the time spent in the area proximal to the mirror (between 0.5 cm and 2.5 cm from the mirror), which may be more relevant for locomotor activity than for aggressive/anxious manifestations, showed no significant differences between groups. The time spent in the area remote from the mirror, a parameter relevant more to anxiety-type manifestations, was significantly increased in the sucrose + insecticide group, which thus seemed to generally avoid aggressive/dominant-type interactions compared to all other three groups (285.3 ± 15.13 vs. 201.06 ± 28.52, *p* < 0.05 vs. control; 285.3 ± 15.13 vs. 167.2 ± 27.93, *p* < 0.01 vs. Fip; and 285.3 ± 15.13 vs. 127.7 ± 25.9, *p* <0.001 vs. sucrose) (Figure 3D).

### 3.4. Oxidative Stress

**Figure 4:**
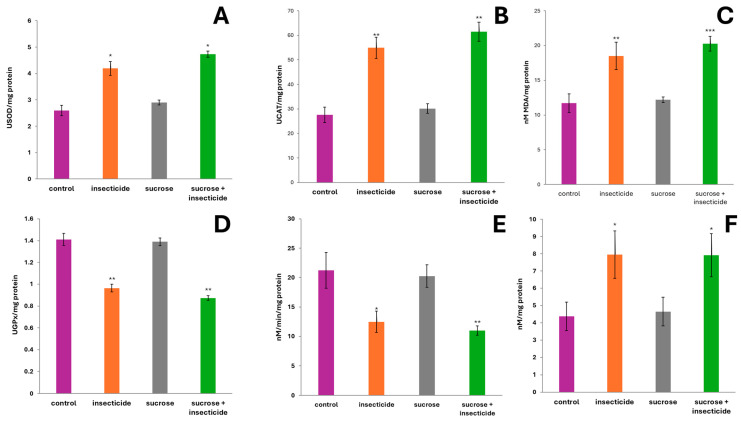
The activity of superoxide dismutase (SOD) (**A**), catalase (CAT) (**B**), lipid peroxidation (MDA) (**C**), glutathione peroxidase (GPx) (**D**), acetylcholinesterase (ACHE) (**E**), and carbonylated proteins levels (**F**) in Silver crucian carp chronically exposed to the chemicals. Data are expressed as the mean ± SD (n = 5), analyzed by one-way ANOVA followed by Tukey’s post hoc test. Statistically significant differences are denoted by * *p* < 0.05, ** *p* < 0.01, and *** *p* < 0.001.

In the present study, we investigated the activities of SOD, GPx, catalase, carbonylated proteins, ACHE, and MDA to assess oxidative stress in Silver crucian carp following chronic exposure to the mixture of insecticide and sucrose. As can be seen in Figure 4, all biochemical indicators of oxidative stress resulted in significant variations (*p* < 0.05 ANOVA).

When cells are exposed to oxidative stress, the production of superoxide radicals increases [56]; considering our results, this was observed only in the two groups exposed to the insecticide or its mixture with sucrose.

SOD is an essential antioxidant enzyme that catalyzes the dismutation of superoxide radicals into hydrogen peroxide and oxygen. The group exposed to the individual insecticide, but also the group treated with the mixture with sucrose, had significantly higher values (*p* < 0.05 Tukey) of SOD (4.1906 USOD/mg protein and 4.7316 USOD/mg protein, respectively) compared to the control group. However, in the case of individual sucrose administration, the SOD level (2.8988 USOD/mg protein) did not show significant differences from the control group. SOD activity usually increases as a protective mechanism to mitigate oxidative damage [57]. Thus, the significant increase in SOD activity in the two groups treated with insecticide and insecticide + sucrose suggests that the body actively responds to a highly oxidative environment, trying to restore the redox balance.

Catalase is an enzyme that catalyzes the breakdown of hydrogen peroxide (H_2_O_2_) into water and oxygen [58]. When oxidative stress occurs, such as after insecticide exposure, hydrogen peroxide levels can increase due to the action of other ROS and SOD activity. The first response to oxidative stress is usually the activation of these antioxidant enzymes. Interestingly, in the insecticide + sucrose group, sucrose seemed to contribute to the oxidative increase triggered by the insecticide: 61.5178 UCAT/mg protein in the insecticide + sucrose group and 55,991 UCAT/mg protein in the insecticide group.

The significant differences observed in CAT activity (*p* < 0.05) compared to the control group suggest that the increase is biologically relevant, not just incidental.

GPx is a critical enzyme that reduces hydrogen peroxides and lipid peroxides using glutathione (GSH) as a cofactor. Decreased GPx activity suggests that the antioxidant defense system is compromised in the presence of a pro-oxidant agent, leading to a reduced ability to detoxify harmful peroxides [57]. Significantly lower levels of GPx indicate that insecticide-induced oxidative stress is overwhelming the enzyme’s ability to function efficiently. This could be due to several factors, including the depletion of glutathione levels, a substance which is required for GPx activity. Prolonged oxidative stress can lead to a reduction in available GSH, affecting GPx function.

Unlike SOD and CAT, which could be upregulated in response to oxidative stress, GPx did not show the same compensatory increase. This could have been due to specific toxic effects of the insecticide on cellular pathways that regulate GPx expression or activity.

The decrease in GPx activity was statistically significant in both the insecticide and sucrose + insecticide groups (*p* < 0.01 vs. control); this strengthens the conclusion that the insecticide negatively affects antioxidant defense mechanisms.

Decreased GPx activity, in combination with increased MDA levels, indicates that cells are experiencing increased oxidative stress and damage [59]. This imbalance suggests that, while SOD and CAT attempt to mitigate oxidative damage, decreased GPx activity may lead to an accumulation of hydrogen peroxide and other peroxides, further contributing to cell damage [60].

MDA is a well-established marker of lipid peroxidation, which occurs when ROS attack polyunsaturated fatty acids in cell membranes [61]. The significant increase in MDA levels indicated that exposure to the insecticide and the mixture of insecticide + sucrose led to oxidative stress that was substantial enough to cause significant cellular damage. The concomitant increase in SOD and CAT activity alongside elevated MDA levels highlighted a complex interplay between oxidative stress and antioxidant defense mechanisms. While SOD and CAT were upregulated to combat oxidative stress, the significant increase in MDA suggests that oxidative damage might have overwhelmed the ability of these antioxidant enzymes to fully neutralize reactive species.

In the context of the group subjected to exposure to sucrose and the insecticide, the malondialdehyde levels were found to be significantly increased (*p* < 0.01), suggesting that sucrose may have enhanced the destabilizing effects associated with the insecticide, indicating a potential synergistic interaction between these two substances which deserves further investigations.

In addition, the insecticide used in this study is known to be a strong inhibitor of the enzyme acetylcholinesterase [62]. ACHE is responsible for breaking down the neurotransmitter acetylcholine (ACh) in the synaptic cleft, thus ending signal transmission between neurons [63]. Decreased ACHE activity indicates that the insecticide binds to the enzyme, preventing it from performing its function. This inhibition leads to a build-up of acetylcholine, which can disrupt normal neurotransmission.

The inhibition of ACHE can lead to the prolonged stimulation of cholinergic receptors due to the accumulation of acetylcholine. This can lead to the overstimulation of the nervous system, which can lead to symptoms such as muscle spasms, spasms, and other neurotoxic effects. Decreased ACHE activity is a direct consequence of the insecticide’s mechanism of action as a neurotoxin.

The decrease in ACHE activity was statistically significant in the insecticide group (*p* < 0.05 vs. control); this reinforces the conclusion that the insecticide has a measurable impact on cholinergic signaling. Regarding the group exposed to insecticide + sucrose, the magnitude of the decrease was even more substantial, with values reaching statistical significance (*p* < 0.01). This result suggests that sucrose has the potential to amplify the disruptive impact of the insecticide.

This finding is particularly important for understanding the neurotoxic effects of the insecticide and its potential implications for animal and human health.

Furthermore, a reduction in ACHE activity may correlate with changes in other biomarkers of oxidative stress and cellular damage. For example, the inhibition of ACHE leads to increased oxidative stress, which could further exacerbate the effects of the insecticide in question on cellular health.

Carbonylated proteins are a reliable indicator of oxidative damage to proteins [64]. The significant increase in protein carbonyls found in our study suggests that exposure to the insecticide may lead to the oxidative modification of the proteins, potentially altering their structure and function. This finding, on the one hand, can be coupled with the increased CAT activity, highlighting the body’s attempt to combat oxidative stress and, on the other, complements the increase in lipid peroxidation.

## 4. Discussions

Continued population growth has created the need to increase agricultural production, namely through the use of pesticides. The systematic administration of said pesticides, on a large scale, has been linked to the emergence of diseases, including neurodegenerative diseases such as Parkinson’s disease (PD), Alzheimer’s disease, or amyotrophic lateral sclerosis [65]. Recent studies claim that exposure to pesticides affects neural circuitry or the differentiation of adipocytes, thus affecting feeding behavior, with the subsequent establishment of obesity, one of the factors involved in the initiation of type 2 diabetes [66,67]. A research study in elderly people living in agricultural areas with a high level of pesticides demonstrated that, due to the inhibition of acetylcholinesterase by the organophosphates present in these pesticides, there was an increased risk of developing AD [68]. While acetylcholinesterase inhibitors are used therapeutically to manage AD symptoms, chronic exposure to organophosphates leads to prolonged cholinergic dysfunction, oxidative stress, and neuroinflammation, contributing to neurodegenerative processes which differ from controlled, short-term therapeutic use.

In the context of experimental toxicology, fish represent an ideal animal model due to their response to environmental toxic compounds, even nanoparticles [69,70]. Silver crucian carp has been widely used to study the toxic effects of insecticides, such as trichlorfon [71], deltamethrin [72], chlorpyrifos [73], thiocarbamate [74], diazinon [75], and hexachlorobenzene [76]. In this study, we used a common insecticide with GABAergic antagonist action in combination with sucrose to assess changes in short-term working memory, anxiety, and aggression behavioral markers in *Carassius auratus gibelio*. The Insecticide group had a higher latency, although not statistically significant, compared to the control group, suggesting a possible anxiogenic effect induced by the insecticide and the potentiation of this effect when combined with sucrose (latency to being in the open, in the dark). The delayed latency to enter the illuminated compartment in the sucrose plus insecticide group reflected heightened anxiety levels. This behavior was consistent with the anxiogenic effects observed in another study, where prolonged exposure to insecticides disrupted GABAergic signaling, leading to CNS hyperactivity and increased stress responses [77]. Sucrose exacerbated this by inducing metabolic stress and oxidative damage, further impairing neurotransmitter regulation and amplifying anxiety-like behaviors. Reluctance to explore the illuminated area indicated an increased aversion to perceived risk, a hallmark of anxiety, corroborated by other observed markers such as reduced exploratory activity and elevated freezing times [78]. The significant decrease in the frequency of entries into the illuminated compartment in the sucrose + insecticide group compared to the sucrose-only group likely arose from the combined neurotoxic effects of insecticides and the metabolic stress induced by sucrose. While sucrose alone may enhance exploratory behavior by providing an energy boost, the addition of insecticides disrupts CNS function, particularly through GABA receptor antagonism, leading to increased anxiety and impaired decision making [77,79]. In our study, this combined effect likely outweighed the influence of sucrose, resulting in reduced exploratory behavior and a preference to avoid the illuminated area, indicating increased anxiety and impaired cognitive function [79]. In addition, the increased frequency of returns to the dark compartment may indicate an enhanced risk-assessment behavior triggered by the anxiogenic effects of insecticides. Thus, this tendency aligns with observations of hypervigilance and repetitive risk-avoidance actions observed in anxiety disorders [80]. In this context, the dark compartment likely served as a perceived safe zone, as heightened anxiety often leads to a preference for environments with less perceived threats [81]. While sucrose may provide a buffering effect against stress by modulating serotonergic signaling [82], the metabolic stress and oxidative damage it induces, especially when combined with insecticides, may amplify avoidance responses.

Also, Abreu et al., in 2019, observed that the exposure of *Carassius auratus* to stressors led to increased anxiety-like behaviors, assessed by changes in swimming and exploration patterns in a novel environment [83]. This study highlighted the relationship between stress and altered behavioral responses, including increased aggression and reduced exploratory behaviors [83]. Overmeyer and his collaborators [84] observed, both at reduced concentrations and at higher concentrations of fipronil, atypical behaviors and muscle control compared to the initial state in aquatic insects from the Diptera family, similar to our results in crucian carps. Thus, the mechanism of action of fipronil involves the disruption of the normal functioning of the central nervous system (CNS) in insects and other arthropods [84]. Specifically, fipronil binds to the GABA receptor, blocking the influx of chloride ions and leading to excitation and convulsions in insects, ultimately causing death at sufficient doses [84,85]. The results of the T-maze test provide key insights into how pesticide exposure, particularly in combination with sucrose, could affect cognitive functions such as short-term spatial memory. Thus, the antagonistic action of fipronil on GABAergic neurotransmission may disrupt the inhibitory signals necessary for proper cognitive processing, as GABA plays a crucial role in modulating hippocampal activity, which is essential for the formation and retention of spatial memory [84,85]. Although sucrose may provide some buffering effects against anxiety through its impact on the HPA axis and serotonergic pathways [86,87], it may also exacerbate neurocognitive dysfunction due to its known metabolic effects. Excessive sucrose intake has been shown to induce hippocampal insulin resistance and oxidative stress, both of which have a negative impact on spatial memory and synaptic plasticity [88,89]. Furthermore, the interaction between insecticide-induced oxidative damage and sucrose-induced metabolic stress could lead to combined effects that affect neurogenesis and memory-related pathways in the brain. As such, the combination of insecticide and sucrose highlights a broader concern about the ecological and environmental implications of pesticide use. Studies in zebrafish have shown that exposure to sublethal concentrations of pesticides can lead to persistent memory and learning deficits [90], suggesting that similar disruptions may occur in other aquatic species.

In the case of the T-test, often used to evaluate learning and spatial memory in animal models (mice, rats, fish, etc.), in this study, we used it to be able to evaluate the anxiety, aggression, and spontaneous exploratory behaviors of carp fish, *Carassius auratus*. In the evaluation of the last parameter, a uniform tendency to explore the three arms was observed for all groups subjected to this study, compared against the control, accompanied by chaotic swimming in the case of the group treated with the insecticide. In addition, exposure to the insecticide led to chaotic swimming behaviors in the treated group compared to the control group, showing a uniform tendency to explore the three arms of the experimental setup. Thus, significant changes in behavior due to insecticide exposure highlight its impact on aquatic organisms’ swimming patterns and general behavior. These aspects were also observed in another study where zebrafish embryos were used, in which the locomotor deficiency characterized by chaotic swimming and spontaneous contractions of the trunk muscles together with explosive bouts of swimming was highlighted [91]. Although in the case of the parameter related to the time spent exploring the three arms no major differences were recorded between the groups, the same cannot be said in the case of the parameter related to the number of entries in the three arms of the device, in which significant differences were recorded for the insecticide group, with a predilection for the central arm, accompanied by chaotic swimming, reinforcing the idea presented in another study by Stehr et al., in which zebrafish exposed to fipronil toxicity for a longer period of time (72 h) showed chaotic swimming due to their capacity for normal movement being affected, alongside undulations of the body, thus suggesting the impossibility of swimming (*p* < 0.001 vs. control) [92].

In Silver crucian carp, the significant drop in alternating tetragram sequences (LRLR, RLRL) observed in the group exposed to sucrose and insecticides could be attributed to the combined effects of neurotoxicity and oxidative stress. Insecticides, such as organophosphates, inhibit acetylcholinesterase, disrupting neurotransmission and impairing memory and decision making processes [93]. Sucrose consumption can exacerbate these effects by inducing metabolic stress and neuroinflammation, which may damage the hippocampus, a region critical for spatial memory and exploratory behaviors [94]. These mechanisms support the link between combined exposure and cognitive dysfunction.

The T-maze can be modified to assess fish anxiety, learning ability, and spatial memory, based on a protocol described by Darlan and Dowling [95]. For the evaluation of spontaneous exploratory behaviors, two parameters were investigated in our experiment, namely the tendency to explore the three arms of the T-maze (left, right, and center) and the number of entries in the three arms. Thus, a slight preference of the insecticide group to explore the left arm over the other two arms was observed, but there were no significant differences between the other groups (*p* < 0.005).

The relationship between anxiety and aggressiveness seems to be influenced by the level of anxiety and may be mediated by various neuronal and hormonal mechanisms [96,97,98]. In our view, the sucrose group entered a state of enhanced arousal linked to the increased anxiety caused by sucrose exposure. Long-term sucrose consumption in animals is reported to produce neuronal alterations of the amygdala [99] or accumbens nucleus [100], which are mesolimbic regions modulating fear responses. Other reports on the effects of the chronic consumption of sucrose in rodents show altered steroid levels and dopamine signaling in the brain, particularly in the mesocorticolimbic system and hypothalamus [101]. Arguably, hyperglycemia may lead to disruptions in these brain circuitries, which have been previously suggested to be associated with impulsivity and violence in humans [102]. Remarkably, a very recent study showed that hyperglycemic rats engaged in hyper-sociable and hyper-aggressive encounters more often than the controls. The key mechanisms underlying this abnormal behavior would include hyperglycemia-induced neuronal oxidative stress damage in brain areas, such as the midbrain, striatum, frontal cortex, and hippocampus [103]. In the case of the insecticide and sucrose group, it is likely that high levels of anxiety—the result of the combined exposure to the insecticide and sucrose—caused a disruption along the stress regulation pathways or other systems that support aggression. In our study, the neurotoxic insecticide altered the GABAergic and glutamatergic neurotransmitter systems, causing reduced exploration and frequent sudden returns to safe areas during the light/dark test. Physiologically, anxiety is related to activating stress responses and circuits—the HPA [104]—which could induce withdrawal and escape responses. Several papers indicate hyperglycemia as eliciting anxiety-like behaviors and robust stress in zebrafish [105]. Significant increases in the levels of cortisol have been shown to be correlated with higher visceral fat deposits, insulin resistance, and increased sugar consumption [106]. Correlations between persistent anxiety and reduced aggression have been reported in the context of elevated stress hormone levels, inhibiting aggression-related circuits in the hypothalamus and amygdala [107].

Following the preliminary analysis, we observed the instillation of a degree of anxiety, especially in the group of fish treated with the insecticide and, to a lesser extent, the group treated with sucrose and the insecticide (this was suggested by the elevated freezing times during the T-test), as well as the significant reduction in exploratory activity in the previously mentioned groups. Similarly, in the light/dark preference test, the assessment of the state of anxiety in the carps showed the anxiogenic effect of the insecticide, potentiated by its association with sucrose, which was consistent with other works in the specialized literature. For instance, Hussain and collaborators observed, in zebrafish, that exposure to pesticides induced more rotational movements in the dark period compared to the light period, relative to other groups [108]. The increased rotational movements observed during the dark period compared to the light period were likely due to fipronil’s disruption of circadian rhythms and altered activity patterns [108,109].

In the mirror-biting test, the main parameters of aggressivity, biting, and frequency of entries into the mirror contact zone were significantly decreased in the insecticide + sucrose group. Audira et al., in 2020, evaluated the aggressive behavior of zebrafish under the action of various toxic pollutants and found less aggressive behaviors in almost all the treated groups [110]. We also observed such an abnormal behavior in our study, which could indicate a synergistic effect of administrating the insecticide together with sucrose. Fipronil, impairing GABA’s role as an inhibitory neurotransmitter, can lead to the hyperactivation of the HPA axis and elevated cortisol levels [111], which, in turn, can impact the functioning of the amygdala and hippocampus [112,113], subsequently exacerbating anxiety responses, including freezing. Excessive dietary sucrose consumption can disrupt the autophagy of hypothalamic neurons and white adipose tissues, leading to an increased caloric intake and weight gain, thereby promoting obesity and the development of metabolic diseases [114]. A high sucrose intake is known to be a factor contributing to the development of metabolic syndrome, including fatty liver [115]. Studies in rats have shown that time-restricted feeding and a high-cholesterol diet, whereby rats are consuming food every six hours, can lead to liver damage, increased cholesterol levels, and weight gain [116]. The lack of freezing in the insecticide–sucrose group can be linked with a protective effect of sucrose along the stress-induced anxiogenesis pathway. Previously, sucrose has been shown to attenuate HPA axis responses to stress in humans and animals [86,117], possibly by activating serotonergic signaling in several reward-regulatory brain sites, which can counteract the effects of stress [87]. Clearly, the interplay between sucrose and fipronil, or other insecticides, suggests more complex aspects to be elucidated by further in-depth studies.

Also, changes in memory were observed in the context of the administration of the combination of insecticide and sucrose, showing the neurodegenerative interaction caused by the insecticide and the metabolic syndrome induced by sucrose. One of the mechanisms underlying the common interaction between neurodegenerative disease and metabolic syndrome could be oxidative stress, manifested both by the production of reactive oxygen species, ROS, or by a reduction in antioxidant capacity, causing damage to the body at the cellular level, more precisely, at the level of its components [118]. Also, glucose homeostasis can be affected by oxidative damage caused by the effect of organophosphates in pesticides [119]. The significant increase in CAT activity in these two groups indicates that the body is responding to high levels of hydrogen peroxide by increasing its ability to neutralize this potentially harmful compound.

In particular, the lack of significant changes in SOD levels in the sucrose-only group may indicate that sucrose does not directly trigger an antioxidant response or activate pathways that regulate SOD expression. This suggests that sucrose alone may not be sufficient to induce a significant protective effect against oxidative stress. However, when combined with insecticides, sucrose may play a role in increasing oxidative damage, leading to an increased SOD response as the body attempts to counteract the higher oxidative load [120]. This finding highlights the differential impact of sucrose on antioxidant defense mechanisms depending on the context. While sucrose alone does not appear to significantly activate SOD, its presence in combination with insecticides appears to amplify the response to oxidative stress, leading to a stronger upregulation of antioxidant enzymes as a compensatory mechanism. Similarly to SOD, increased CAT levels can be viewed as a compensatory response to oxidative stress. The first response to oxidative stress is usually the activation n of these antioxidant enzymes. When cells detect higher levels of ROS, including hydrogen peroxide, they can upregulate the expression of antioxidant enzymes such as catalase to protect against oxidative damage. This adaptive response helps maintain cellular homeostasis and prevent oxidative damage [121]. The pronounced increase in SOD and catalase activity observed in the insecticide + sucrose group indicates that the organism actively responds to the increased OS by upregulating its antioxidant defense mechanisms. This suggests that these groups were subjected to a significant oxidative load, leading to a strong adaptive response to neutralize ROS and mitigate potential cellular damage. The increased catalase activity, especially in the insecticide + sucrose group, indicates an intensified response, probably determined by the combined effects of insecticide and sucrose. Thus, we can say that sucrose may contribute to an exacerbated oxidative environment, requiring a more robust activation of antioxidant enzymes [122]. The upregulation of both SOD and catalase reflects an adaptive mechanism aimed at counteracting oxidative damage and preserving cellular redox balance. However, the marked increase in enzymatic activity suggests that the organism is subjected to considerable OS. Although these adaptive responses are protective, they may not be sufficient to completely prevent cellular damage, which can lead to long-term repercussions for cellular and systemic health.

To sum up, these results indicate that decreased GPx activity and increased MDA levels were clear indicators of increased oxidative stress and cell lesions in the insecticide and sucrose + insecticide groups. The production of ROS leading to molecular damage and potentially favoring behavioral dysfunctions was likely due to specific toxic effects of the insecticide on cellular pathways that regulate GPx expression or activity. Elevated MDA levels, as a marker of lipid peroxidation, reflected significant damage to cell membranes caused by OS. This membrane damage could disrupt cellular signaling and neuronal integrity, critical for normal brain function. Weakened antioxidant defense systems, as indicated by reduced GPx activity, could have broader physiological consequences beyond neuronal damage. For example, OS can impair mitochondrial function, leading to reduced energy production and increased apoptosis, which are critical for tissue homeostasis [123,124,125]. Additionally, oxidative damage to the DNA, proteins, and lipids can contribute to systemic inflammation and accelerate aging processes, potentially predisposing organisms to chronic diseases such as neurodegeneration, cardiovascular disorders, and metabolic dysregulation [126,127]. In the pesticide and insecticide + sucrose groups, the oxidative damage might have interfered with synaptic transmission and neuronal plasticity, leading to alterations in the neural circuits associated with anxiety and aggression [128]. Behavioral manifestations such as increased anxiety or aggression could have resulted from damage to the brain regions involved in emotional regulation, such as the amygdala and the prefrontal cortex, where OS might have impaired neurotransmitter balance or receptor functionality [129,130].

However, the group in which oxidative stress had the highest values in terms of harmful oxidative stress byproducts and the lowest values in ACHE enzymatic activity was the insecticide–sucrose group, which suggests that sucrose may function as an exacerbating or even pivotal factor in heightening the susceptibility of organisms to environmental stressors. Within this specific group, there was not only a noted compromise in antioxidant defense mechanisms, but also the emergence of more pronounced behavioral deficits of a cognitive or affective nature, which were absent in the control group. The observed behavioral deficits, such as those recorded in the T-maze and mirror-biting tests, might have been directly linked to the decrease in ACHE activity. In the T-maze, reduced ACHE activity and subsequent acetylcholine accumulation could have impaired the cholinergic pathways critical for learning and memory, resulting in disorganized behavior or diminished cognitive performance [131]. In the mirror-biting test, the overstimulation of cholinergic receptors due to inhibited ACHE may have led to increased impulsivity, heightened anxiety, or hyperactivity. These behavioral changes reinforce the hypothesis that the neurotoxic effects of the insecticide, amplified by sucrose, disrupt normal cholinergic signaling and contribute to the observed cognitive and affective impairments. Additional explorations are needed regarding the mechanistic interactions between the insecticide and sucrose.

## 5. Conclusions

In conclusion, the administration of the insecticide alone as well as its combination with sucrose had anxiogenic effects, disrupting the normal behavior of carp fish. Specifically, fish exposed to the insecticide alongside sucrose demonstrated a significant reduction in exploratory activity and aggressive behaviors. Furthermore, this combination adversely affected memory and spatial learning in the fish, suggesting a potential neurodegenerative interaction. The insecticide used operates by binding to GABA receptors, blocking the influx of chloride ions, and leading to excitation and convulsions in insects. This same mechanism appeared to affect fish, disrupting CNS function. Therefore, while we can discuss interactions between the insecticide and sucrose, it is crucial to clarify that the observed effects indicate a complex relationship rather than a strictly synergistic one. Additionally, a high sucrose intake can contribute to metabolic syndrome, including fatty liver and oxidative stress, which can be exacerbated by the effects of pesticides such as the insecticide used in this study. Our findings indicate that oxidative stress levels were significantly elevated in the group exposed to both the insecticide and sucrose compared to the control group, highlighting the potential toxicity of this combination.

This research underscores the detrimental impact of co-exposure to the insecticide and sucrose, suggesting that such interactions may lead to irreversible cellular damage. Nevertheless, further investigations into their combined toxicity are warranted. Future research should focus on elucidating the cellular mechanisms of toxicity and exploring a wider range of concentrations. This highlights the need for further research into the mechanistic interactions between insecticide and sucrose. In conclusion, we can say that pesticide exposure and a high sucrose intake could have significant implications for human health, especially in terms of neurodegenerative diseases and metabolic disorders. Further research is needed to understand these interactions and their effects on human health.

These findings suggest that the interaction between neurotoxins and dietary factors such as sucrose may have broad consequences on cognitive and behavioral functions in aquatic ecosystems and, potentially, in other organisms exposed to these compounds. Further research should explore the molecular mechanisms underlying these interactions, focusing particularly on OS pathways, changes in neurotransmitter signaling, and the potential protective effects of dietary modifications or antioxidant interventions.

## Data Availability

The datasets used and/or analyzed during the current study are available from the corresponding author upon reasonable request.
